# Risk Factors, Manifestation, and Awareness of Osteoporosis among Patients of Various Specialists in Switzerland: Results of a National Survey

**DOI:** 10.3390/healthcare10020295

**Published:** 2022-02-03

**Authors:** Sigrid Jehle-Kunz, Hans-Jörg Häuselmann, Mitra Keschawarzi, Olivier Lamy, Franck Luzuy, Natalie Marcoli, Christian Meier, Brigitte Uebelhart, Peter Wiedersheim

**Affiliations:** 1 Center for Osteoporosis, St. Anna Clinic, 6003 Lucerne, Switzerland; 2 Center for Rheumatology and Bone Diseases, 8038 Zurich, Switzerland; hjhauselmann@rheumazentrum.ch; 3 Mylan Pharma GmbH (a Viatris Company), 6312 Steinhausen, Switzerland; mitra.keschawarzi@viatris.com; 4 Service of Internal Medicine, Department of Medicine, Lausanne University Hospital, University of Lausanne, 1011 Lausanne, Switzerland; olivier.lamy@chuv.ch; 5 Gynecology and Obstetrics, Clinic Grangettes-Hirslanden, 1224 Geneva, Switzerland; f.luzuy@deckpoint.ch; 6 Department of Rheumatology, Regional Hospital of Lugano, 6962 Viganello, Switzerland; natalie.marcoli@eoc.ch; 7 Division of Endocrinology, Diabetology and Metabolism, University Hospital, University of Basel, 4031 Basel, Switzerland; christian.meier@unibas.ch; 8 Division of Bone Diseases, Geneva University Hospitals and Faculty of Medicine, University of Geneva, 1205 Geneva, Switzerland; brigitte.uebelhart@hcuge.ch; 9 FMH Rheumatology, 9006 St. Gallen, Switzerland; peter.wiedersheim@hin.ch

**Keywords:** osteoporosis, risk factors, fragility fractures, supplements, survey, Switzerland, general practitioners, endocrinologists, rheumatologists, gynecologists

## Abstract

Osteoporosis is the most common chronic metabolic bone disease, known to be underdiagnosed and undertreated in parts of the Swiss population. Due to expected rise in new fragility fractures, adequate awareness of associated risk factors and diagnostic and therapeutic options will be essential for the management of osteoporosis. We therefore explored these aspects in a nationwide survey of Swiss specialists and their patients. A total of 262 physician questionnaires and 9065 patient questionnaires were analyzed, mainly from general practitioners (64.9%), followed by rheumatologists (16.8%), gynecologists (12.2%), and endocrinologists (6.1%). Around 20% of patients were under medication and/or had a medical condition increasing the risk of osteoporosis. Further risk factors, such as low consumption of calcium-rich foods, smoking, elevated alcohol intake, and insufficient physical activity, were present across regions and medical fields. 53.9% of patients did not take calcium/vitamin D supplements; 3.5% reported having fragility fractures, and 7.3% received treatment for osteoporosis. Only 38.5% of surveyed patients knew of the chronic nature of osteoporosis, indicating rather low awareness in this population. Despite generally perceived relevance of osteoporosis for daily practice, aspects of its prevention and management varied across regions and medical fields. Raising awareness among patients and physicians will be vital for addressing osteoporosis on a national scale.

## 1. Introduction

Osteoporosis is a growing health concern worldwide, with complications as prevalent as those of other common chronic diseases, such as hypertension and diabetes [1]. It is the most common chronic metabolic bone disease and is defined as a progressive systemic skeletal disease characterized by low bone mass and microarchitectural deterioration of bone tissue leading to an increase in bone fragility and susceptibility to fractures [2,3,4]. Diagnostic criteria for osteoporosis have been defined by the WHO on the basis of bone mineral density (BMD), under which osteoporosis is characterized by a BMD of 2.5 standard deviations or more below the average value for young healthy women [5]. Various risk factors have been identified for osteoporosis, including age, gender, a prior fracture, family history of fractures, and lifestyle-related risk factors such as physical inactivity and smoking [5]. Its occurrence is four times more common in women than in men, although some evidence suggests men are more prone to osteoporosis-related complications [1]. Taken together with advanced age as another major risk factor, the population most commonly affected by osteoporosis are consequently post-menopausal women [6].

Fractures associated with osteoporosis affect various parts of the skeletal system and can cause a substantial burden on patients’ wellbeing, even impacting life expectancy, particularly in case of hip fractures. A study based on Swedish patient register data revealed that 17–32% of all deaths associated with hip fractures were directly or indirectly related to the hip fracture event [7]. Appropriate diagnosis and treatment of osteoporosis nevertheless remained issues yet to be adequately tackled across different countries including Switzerland. Indeed, one in two women and one in five men in Switzerland are expected to sustain a fragility fracture, i.e., a fracture without adequate trauma, after the age of 50 [8]. A previous nationwide survey in Switzerland revealed a diagnosis and treatment gap in Swiss patients aged 50 years and above presenting with a fragility fracture to the emergency ward of participating hospitals [9]. Dual-energy X-ray absorptiometry (DXA) measurement was only performed in 31.4% of patients during the diagnostic workup. Furthermore, only 24% of women and 13.8% of men were adequately treated with a bone active substance, generally an oral bisphosphonate, with or without calcium/vitamin D supplements. This finding was confirmed by a comparison of sales data for osteoporosis medication with the number of individuals eligible for osteoporosis treatment in Switzerland, which found 36% of men and 58% of women with high risk of fractures were not actively treated [10]. As part of that study, a model estimating the clinical and economic burden of osteoporotic fractures based on literature regarding fracture incidence and related costs suggested an approximately 34% increase in new fragility fractures should be expected between 2010 and 2025, accompanied by a cost increase of 29% to 2642 million CHF. Relevance of osteoporosis prevention is further illustrated by findings showing that osteopenia might be present in more than 1/5 of individuals between the age of 35 and 50 regardless of gender [11]. Importantly, osteopenia and osteoporosis represent a continuum, and in absolute terms more patients with osteopenia experience a fragility fracture than patients with osteoporosis, as osteopenia is far more common [12].

For individuals at risk or with manifested osteoporosis, different dietary and lifestyle changes can be introduced to prevent fractures. Within the European Union, use of calcium and vitamin D supplements could prevent more than 500,000 fractures per year in adults with osteoporosis [13]. Prerequisite for successful prevention and management of osteoporosis is adequate awareness of the disease and its risk factors. Improving knowledge on screening as well as general lifestyle and medical measures for bone health and fracture prevention has previously been advocated for in fracture prevention strategies for older individuals [14]. Lack of awareness concerning the disease and its consequences has been identified as an obstacle for widespread use of drugs with antifracture efficacy [15]. As knowledge gaps regarding osteoporosis prevention and management persist in the general population and different stakeholders within the health care system [16,17,18], raising awareness on osteoporosis, its risk factors and prevention will be an important step in shaping future health care policies.

Aim of the present survey was to explore the risk factors, manifestation, and awareness of osteoporosis in Swiss patients, as well as the diagnostic and treatment approaches of their physicians working in private practice. The survey was performed within medical fields most likely to encounter patients in need of osteoporosis prevention or treatment.

## 2. Materials and Methods

A patient and a physician questionnaire were developed and utilized for a nationwide Bone Health Awareness (BHA) survey in Switzerland in order to generate representative field data on aspects of prevention, diagnosis, and treatment of osteoporosis (complete questionnaires in German, Italian and French are provided as Supplementary Materials, S1–S6). Relevant questions were defined by the authors of the present publication as part of their interdisciplinary Advisory Board Meeting covering the fields of rheumatology, endocrinology, internal medicine and gynecology. Risk factors covered by the patient questionnaire are similar to those considered in previous research articles on osteoporosis [19,20]. General recommendations for prevention of osteoporosis and associated fractures outlined by the *Dachverband Osteologie (*DVO) were also taken into account in the questionnaire design [21]. The fracture risk assessment tool (FRAX), as one of the established tools available to physicians for osteoporosis risk assessment, was among the diagnostic approaches covered by the physician questionnaire [22,23]. Regarding their nutrition, patients were asked to specify whether their intake of products listed on the survey (among them common sources of calcium) fell short of or exceeded 7 portions per week. Smoking was reported via a question with answers “yes”, “no”, or “other”, without specifying amount/frequency.

The questionnaires were distributed to general practitioners (GPs), rheumatologists, endocrinologists, and gynecologists across Switzerland, in the language of the given region (German, French or Italian), with relevant fields having previously been specified by the authors. Physicians were selected based on their medical field through a potential contacts’ registry of Mylan Pharma GmbH and approached by the company regarding possible participation in the survey. Each physician expressing interest in participation received one physician questionnaire and 50 patient questionnaires in March 2019, carrying a unique identification number. Physicians were encouraged to reach out to their patients, perform the survey in the week of 25–29 March 2019, and subsequently mail completed physician and patient questionnaires directly to the biometric institute *G.E.M. Gesellschaft für Evaluation und Qualitätssicherung in der Medizin mbH* in Meerbusch, Germany, in order to uphold highest possible standards of anonymity and neutrality during data handling and analysis. Participation in the survey was voluntary and not attached to any financial compensation. In order to obtain representative field data on osteoporosis risk factors, prevention and treatment, physicians could approach all patients from their respective practice without pre-selection based on patients’ osteoporosis status.

**Figure 1:**
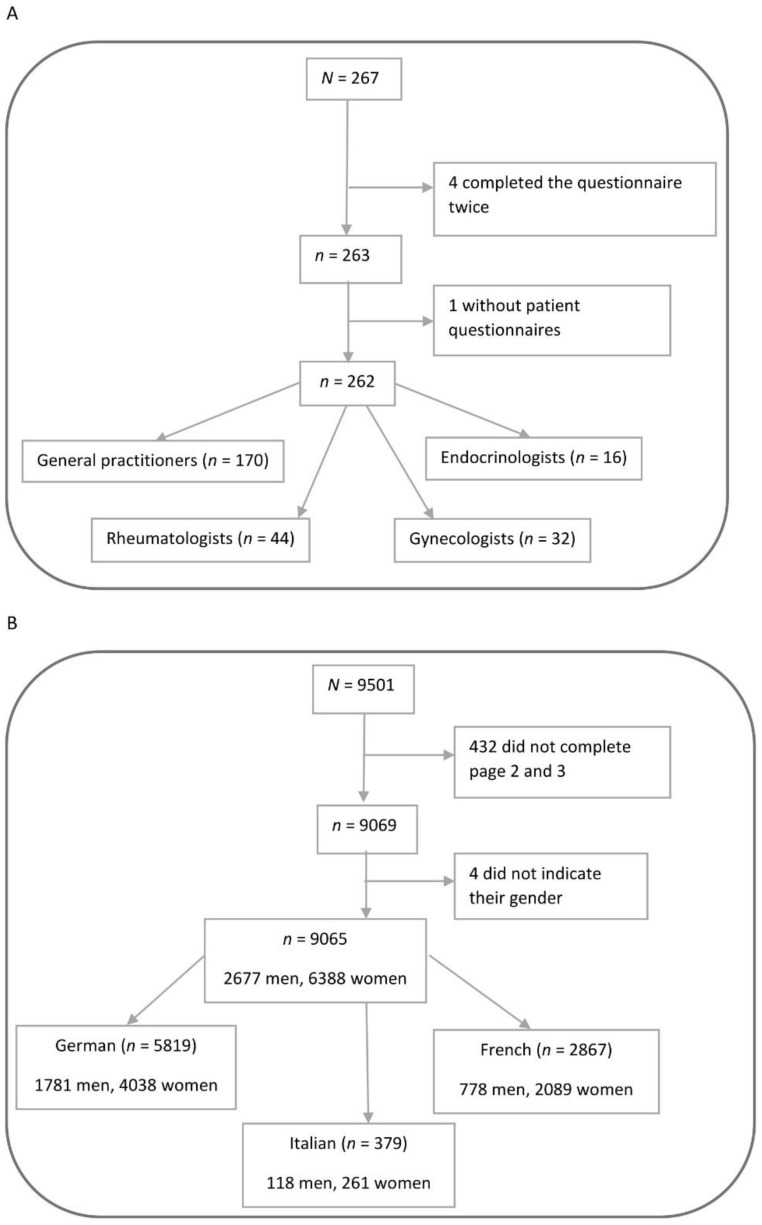
Physician (**A**) and patient (**B**) questionnaire flow.

Completing more than one copy of the physician questionnaire or failing to submit any completed patient questionnaires were regarded as reasons for omission from analysis (Figure 1). Incomplete patient questionnaires lacking relevant details were also excluded. Exploratory analysis included descriptive statistics of all parameters covered by both questionnaires. For categorical parameters, corresponding sample size and frequency distributions were determined. Analysis of continuous or quasi-continuous (ordinal) parameters included the respective sample size, arithmetic mean, and standard deviation and minimal, maximal, and median values. Subpopulation with fragility fractures was comprised of patients ≥50 years of age who reported atraumatic fractures (without external influence as in case of a fall or an accident) that occurred at an age ≥50 years. For easier readability, images are presented without missing values, as their proportion was generally marginal.

## 3. Results

Survey questionnaires were completed by 267 physicians and 9501 patients. Based on the specified criteria, 262 physician and 9065 patient questionnaires (98.1% and 95.4%, respectively) were included in the analysis (Figure 1), with an average of 35.5 ± 14.2 patient questionnaires per physician (GPs: 36 ± 13.4, rheumatologists: 33.9 ± 15.7, gynecologists: 38.6 ± 13.8, endocrinologists: 27.7 ± 17.1). Participating physicians were predominantly GPs (64.9%), followed by rheumatologists (16.8%), gynecologists (12.2%), and endocrinologists (6.1%). The proportion of rheumatologists was higher than in the Swiss outpatient sector comprised of the physician groups targeted by the survey (16.8% vs. 1.5%, respectively; Table 1), whereas the proportion of the remaining specialists was comparable [24]. Regional distribution of surveyed physicians and patients (Table 1) reflected the language distribution in Switzerland [25]. However, none of the participating gynecologists or endocrinologists were from the Italian-speaking region.

### 3.1. Patient Survey

#### 3.1.1. Demographics

**Figure A1:**
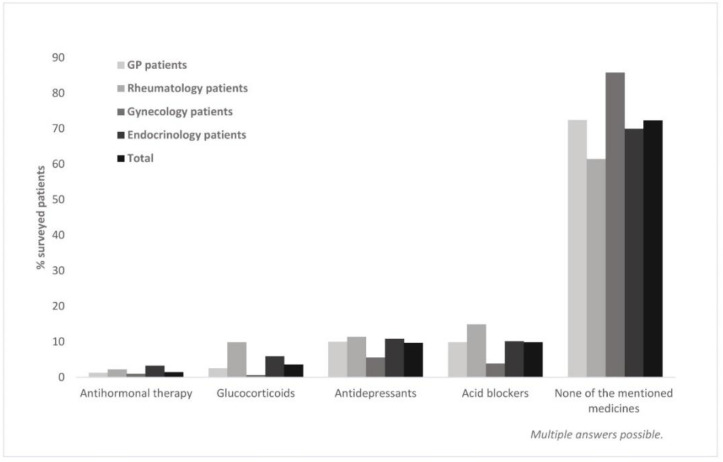
Use of medication associated with an increased risk of osteoporosis among the surveyed patients.

**Figure A2:**
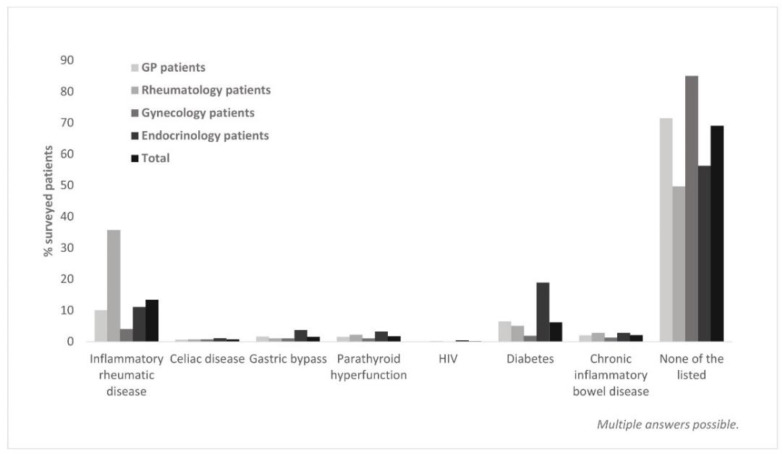
Medical interventions and conditions associated with an increased risk of osteoporosis among the surveyed patients.

Surveyed patients were predominantly female (70.5%). Mean age was 53.1 years, somewhat above the Swiss average of 42.6 years [26], with 28.5% of the patients ≥65 years of age. 38.6% of female patients were menopausal, out of which 17.5% received hormone replacement therapy. 20.9% of patients were under medication associated with an increased risk of osteoporosis (Figure A1). Among rheumatology patients, 9.9% were treated with glucocorticoids. Medication with potential impact on bone health was more frequently reported by patients with fragility fractures (37.2% vs. 25.8% of the comparison group). 22.7% of surveyed patients had a medical condition or intervention associated with osteoporosis (Figure A2). The percentage of these patients was particularly high among rheumatologists (42.2%), and lowest among gynecologists (9.1%). 38.8% of patients with fragility fractures had at least one specified risk condition, vs. 29.2% of patients in the comparison group. Patients with fragility fractures more frequently reported a hip fracture in close relatives, somewhat above survey average (Table 2). Percentage of endocrinology and rheumatology patients as well as patients from the Italian-speaking region reporting a family history of hip fractures was also above-average (Table 2). In contrast, the percentage of gynecology patients with respective family history was markedly below average.

#### 3.1.2. Nutrition and Lifestyle

**Figure 2:**
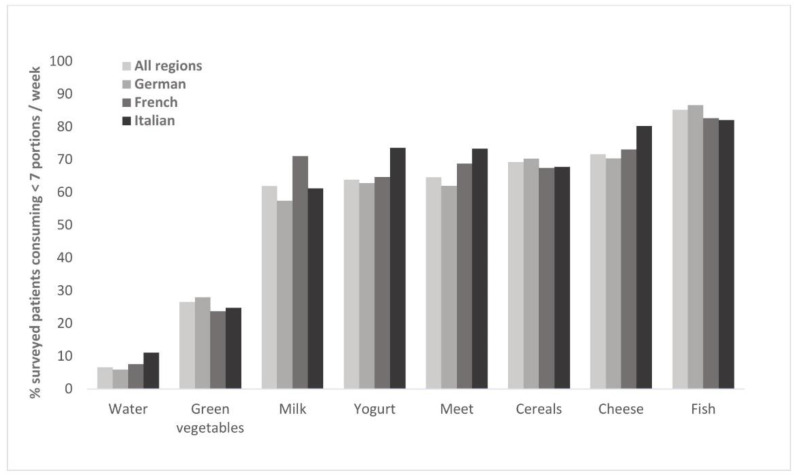
Recorded intake of calcium-rich products and meat.

**Figure A3:**
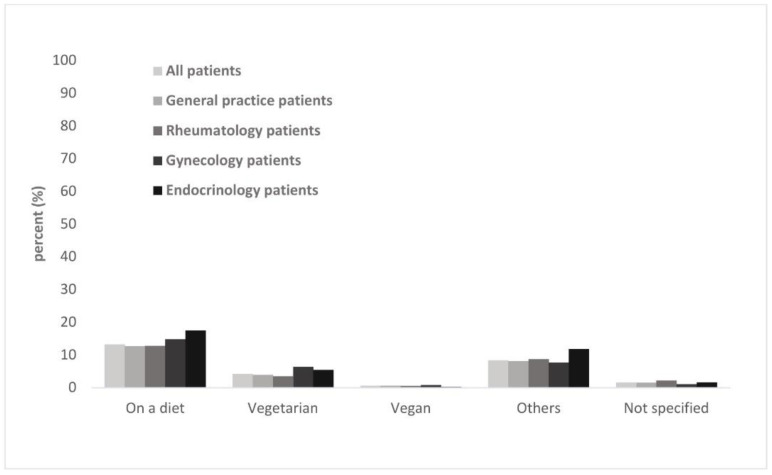
Dietary habits of the surveyed patients.

A rather small proportion of patients (13.2%) reported following a certain diet, mostly other than vegetarian or vegan (8.4%). 4.3% were vegetarian, and only 0.6% vegan. Regarding the nationwide consumption of calcium-rich products, the intake of water and green vegetables was relatively high, and the intake of dairy products relatively low (s. Figure 2). Above-average proportions of patients on a diet were found with gynecologists and endocrinologists (14.8% and 17.5%, respectively, s. Figure A3).

**Figure 3:**
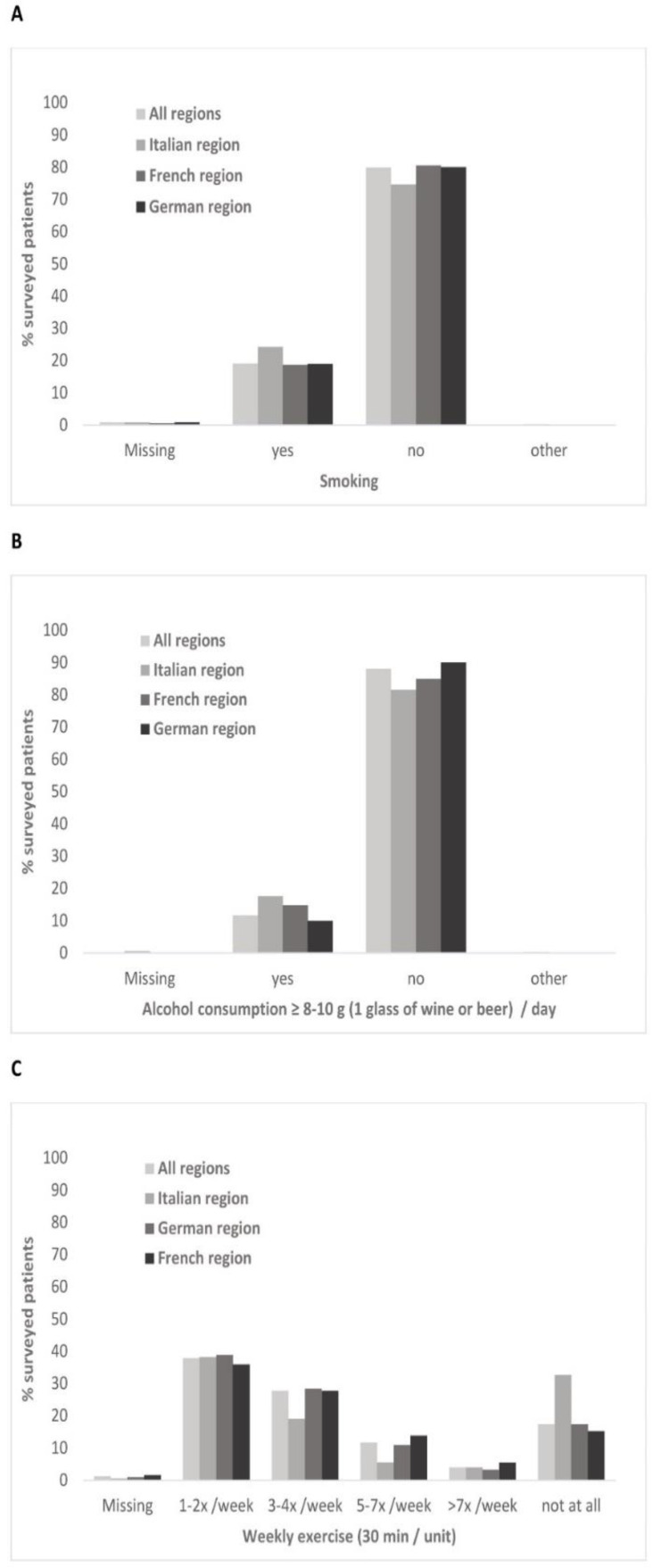
Lifestyle habits of surveyed patients: (**A**) smoking; (**B**) alcohol consumption; (**C**) weekly exercise.

Surveyed patients were predominantly non-smokers (80%), without an elevated alcohol intake of more than one glass of wine or beer per day (88.1%, s. Figure 3A,B). Most patients (81.5%) reported they exercised at least 1–2 x per week for 30 min, with some apparent regional differences (Figure 3C). Somewhat more patients with fragility fractures reported not engaging in sports than in their comparison group (22.1% vs. 16.6%).

#### 3.1.3. Supplementation with Calcium/Vitamin D

**Figure 4:**
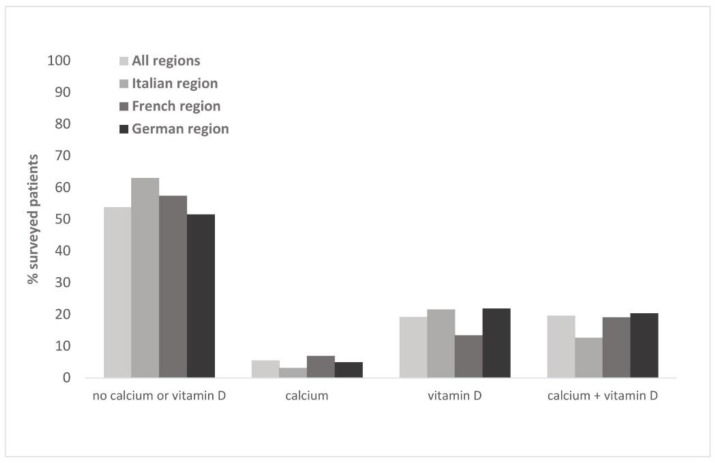
Intake of calcium/vitamin D supplements.

**Figure A4:**
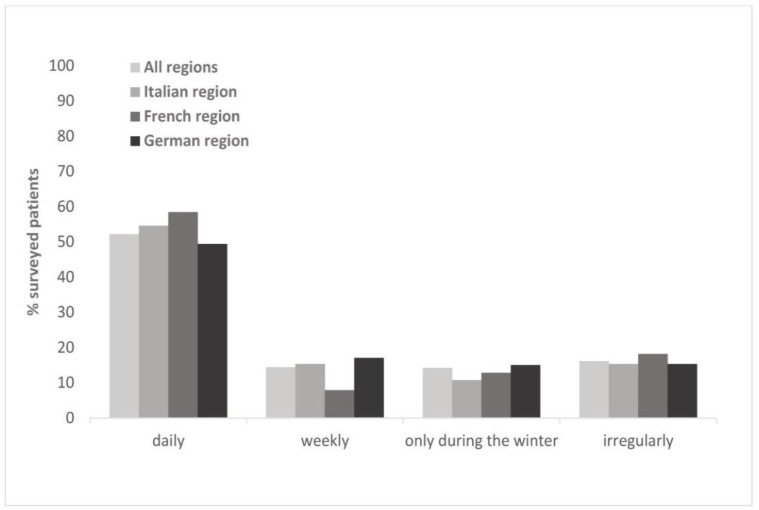
Frequency of calcium and/or vitamin D supplementation.

More than half of the patients (53.9%) reported not taking any calcium/vitamin D supplements (Figure 4). Most patients with supplement intake used both calcium and vitamin D supplements (19.7%), or vitamin D only (19.3%). Supplements were taken predominantly daily (52.2%, Figure A4). Less than 20% of the patients took their supplements on a weekly basis, only in winter or irregularly. Daily intake was somewhat more, and weekly intake less frequent among patients of the French-speaking region. The Italian-speaking region had the lowest proportion of patients with supplement intake (34.3%) and a comparatively low rate of calcium + vitamin D supplementation (12.7%). The rate of supplement intake varied among patients of different physicians in a range between 40% and 56.3% (s. Table 3). 66.6% of patients with fragility fractures used supplements, predominantly calcium and vitamin D.

#### 3.1.4. Bone Health

**Figure 5:**
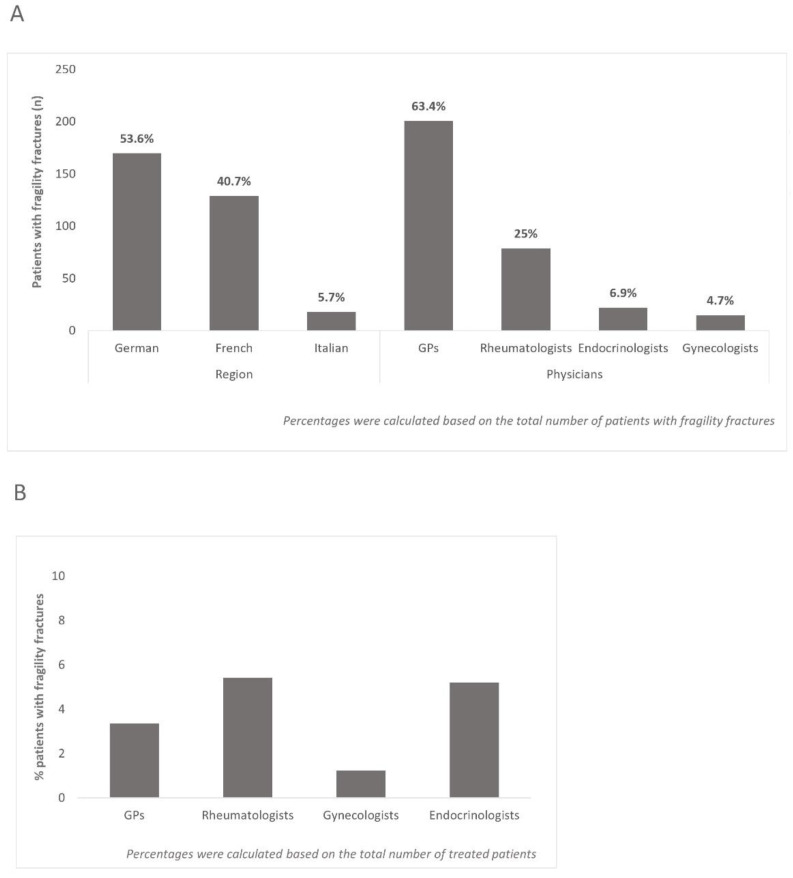
Distribution of patients with fragility fractures across regions (**A**) and medical fields (**A**,**B**).

3.5% of the total patient population reported having at least one fragility fracture. The highest prevalence of fragility fractures, relative to the number of participants from a given region, was found among patients from the Italian-speaking region (4.8%, vs. 4.5% from the French-, and 2.9% from the German-speaking region). This result is notable, as the lowest number of patients came from the Italian-, and the highest from the German-speaking region. Based on the number of patients with fragility fractures in a given region relative to the total number of patients with fragility fractures in the overall sample, highest percentage came from the German-speaking region, and lowest from the Italian-speaking region (Figure 5A). Most patients with fragility fractures were found with GPs, the fewest with gynecologists (Figure 5A). However, based on the number of treated patients, the highest percentage of patients with fragility fractures was found with rheumatologists, closely followed by endocrinologists (Figure 5B). There were some notable differences regarding the evaluation and treatment of fragility fractures reported by the patients compared to usual practices indicated by the physicians, e.g., most patients reported an X-ray and most physicians a bone density measurement (DXA) occurring after a fragility fracture (s. Table 4). In contrast, only 37.5% of patients with fragility fractures indicated having a DXA measurement performed. Contrasting findings were also apparent for the reported assessment of fracture risk, with more than a 10-fold difference between patients and physicians.

**Figure 6:**
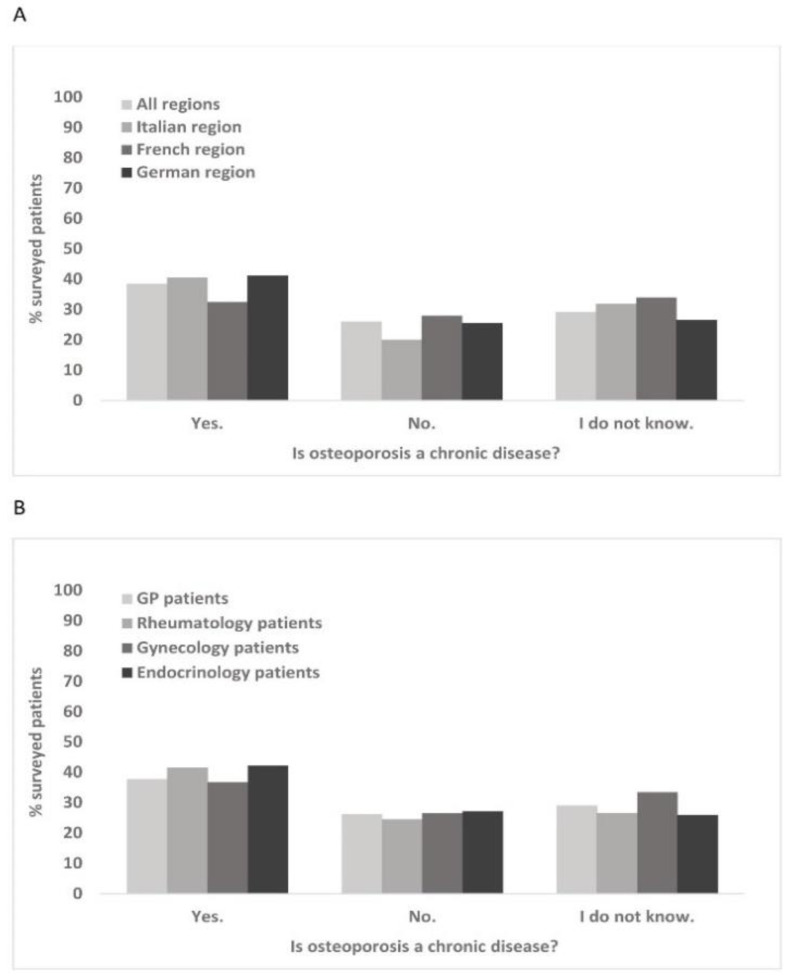
Awareness of the chronic nature of osteoporosis across different regions (**A**) and with patients of various specialists (**B**).

7.3% of the surveyed patients reported receiving treatment for osteoporosis, with relatively similar proportions across different regions. By far the highest percentages of patients on osteoporosis treatment were found with rheumatologists and endocrinologists (14.5% and 13.2%, respectively, vs. 6% of GPs and 3.1% of gynecologists). Only 38.5% of the surveyed patients were aware that osteoporosis is a chronic disease. Indeed, 26.1% of patients even answered the corresponding question with “no” (Figure 6A). Low awareness of the chronic nature of osteoporosis was similar across all regions and physician groups (Figure 6B) and was not influenced by the presence of fragility fractures (data not shown). Most patients were not concerned about bone fragility (84%) and did not feel insecure while walking or afraid of falling (82.1%).

### 3.2. Physician Survey

#### 3.2.1. Osteoporosis in Daily Practice

**Figure 7:**
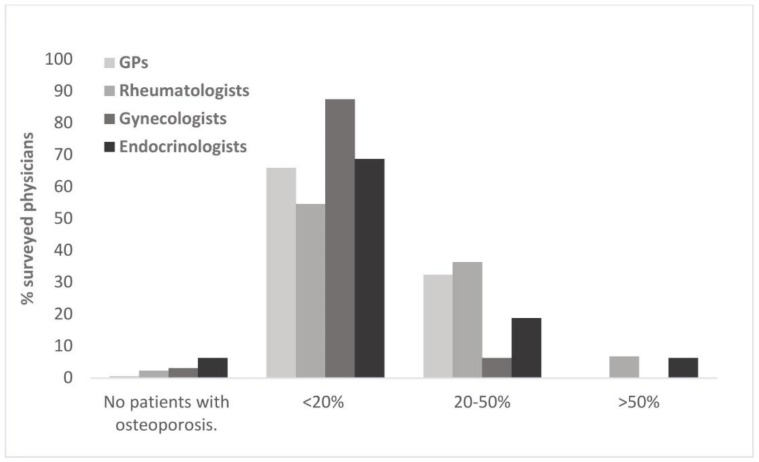
Proportion of treated osteoporosis patients as estimated by different specialists for their practice.

Rheumatologists indicated the highest proportion of osteoporosis patients in their practice, followed by GPs, endocrinologists, and gynecologists (Figure 7). 87.5% of gynecologists indicated the proportion of osteoporosis patients in their practice was <20%. Physicians were also asked to rate the importance of osteoporosis for their daily practice on a scale of 0 to 5, with 5 being the lowest rating (“not important”). The rating was generally high across all regions, with an average value of 1.6. In accordance with the respective proportion of patients with osteoporosis, highest average rating was given by rheumatologists (1.2), followed by GPs and endocrinologists (each with an average rating of 1.6). Lowest average rating was given by gynecologists (2.0).

#### 3.2.2. Criteria for an Osteoporosis Work-Up

Physicians based their decision for an osteoporosis work-up on clinical criteria (89.7%), nutritional deficit (58.4%) or screening (64.1%). In addition, 50.4% of the physicians, most commonly rheumatologists (59.1%), indicated they based their decision on patient initiative. Patient initiative was also taken into account by a considerable proportion of GPs (50%), gynecologists (46.9%), and endocrinologists (37.5%). While the clinical criteria had a similarly high standing across the regions, there were some notable regional differences concerning the remaining criteria e.g., screening was reported by 59% of physicians from the German-speaking, 66.7% from the Italian-speaking and 73% from the French-speaking region. Furthermore, nutritional deficit was considered by 60.3% from the German- and 57.3% from the French-speaking region vs. 41.7% from the Italian-speaking region. Approximately half of the physicians from the German- as well as the French-speaking region indicated patient initiative as a criterion, in contrast to a smaller proportion of 41.7% from the Italian-speaking region.

#### 3.2.3. Evaluation and Treatment of Patients with Fragility Fractures

**Figure 8:**
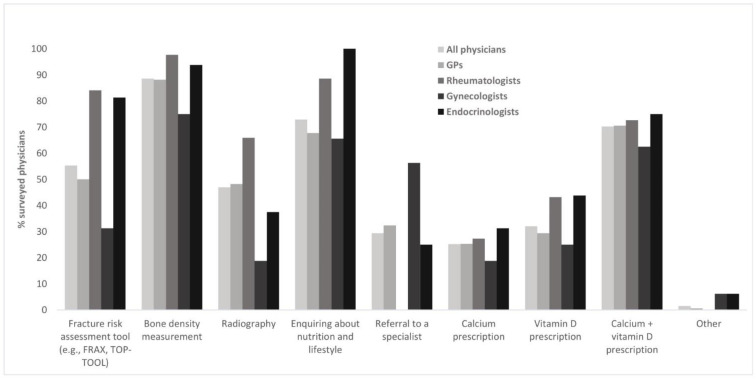
Evaluation and treatment of fragility fractures.

When consulted by patients with fragility fractures, 88.6% of the surveyed physicians would perform a bone density measurement (Table 4), and around 70% would enquire about nutritional and lifestyle habits and/or prescribe calcium and vitamin D supplements. Radiography and the use of risk assessment tools such as FRAX or TOP-TOOL appear less common, as they were reported by approximately half of the physicians (Table 4). Around 1/3 of the physicians indicated prescribing supplements containing only calcium or vitamin D and/or referring the patients to a specialist (Table 4). Regional differences were particularly striking concerning the use of assessment tools and radiography. Use of assessment tools was approximately 20% higher in the French-speaking (60.7%) vs. the Italian-speaking region (41.7%), with the German-speaking region approximately in the middle of the range (53.4%). In contrast, use of radiography showed the opposite pattern (French-speaking: 46.1%, Italian-speaking: 66.7%, German-speaking: 46%). Utilization of these two methods also differed strongly depending on physician specialty, gynecologists being least likely to use them (Figure 8). All endocrinologists, and most rheumatologists, GPs, and gynecologists indicated enquiring about the patients’ nutrition and lifestyle habits. Gynecologists were most likely to refer patients with fragility fractures to a specialist, whereas none of the rheumatologists indicated a referral. Moreover, only 40.6% of gynecologists reported feeling confident in treating patients with osteoporosis, compared to around 3/4 to 4/5 of the remaining specialists (GPs 75.3%, rheumatologists 81.8%, endocrinologists 81.3%). 18.8% of gynecologists indicated they were not trained for the treatment of osteoporosis. In contrast, only 3.5% of GPs and no rheumatologists or endocrinologists reported not being trained.

#### 3.2.4. Prescription of Calcium/Vitamin D Supplements

**Figure 9:**
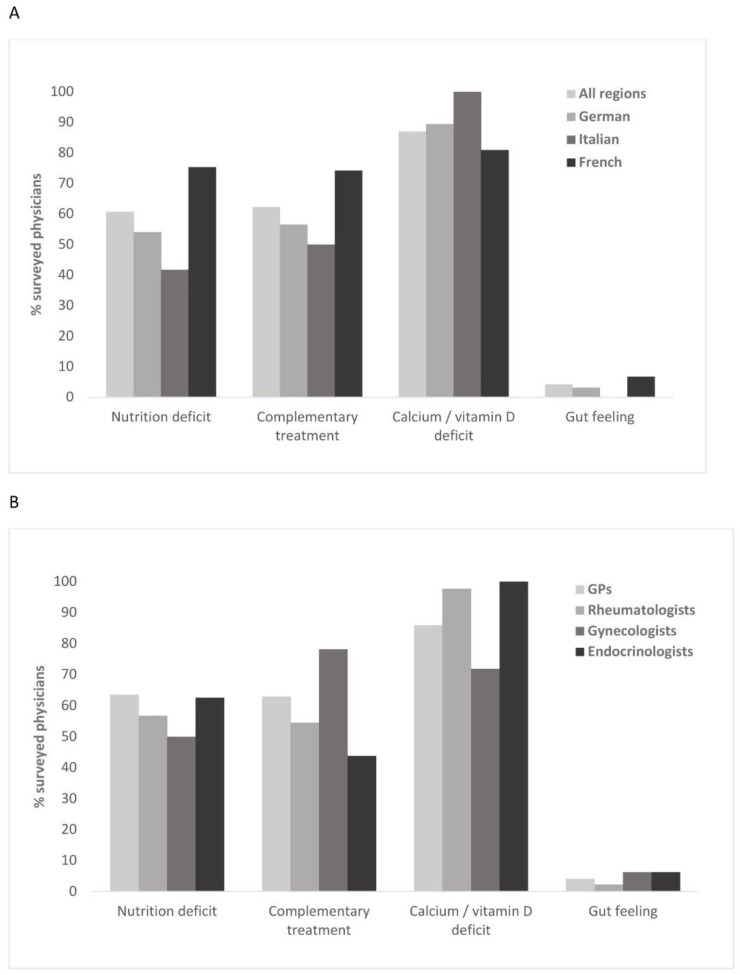
Reasons for prescribing calcium/vitamin D supplements in different regions (**A**) and medical fields (**B**).

87% of physicians prescribed calcium/vitamin D supplements based on an established deficit, whereas approximately 60% indicated a nutrition deficit and/or complementary treatment as criteria, with notable regional differences (s. Figure 9A). The proportion of physicians with nutrition deficit and complementary treatment as criteria was 20–30% higher in the French- as compared to the German- and Italian-speaking region. Moreover, all physicians of the Italian-speaking region indicated calcium/vitamin D deficit as criterion, in contrast to around 4/5 from the French-speaking region. This criterion was also most frequently reported by endocrinologists, and least often by gynecologists (s. Figure 9B). Indeed, gynecologists were the only physician group, in which complementary treatment even slightly superseded an established deficit as a criterion for supplement prescription. The smallest proportion of physicians reporting complementary treatment as a criterion was among the endocrinologists. Among the remaining specialists, nutritional deficit and complementary treatment were otherwise comparable, with a rate of approximately 50–60%.

## 4. Discussion

In our present article, we report on the nationwide Bone Health Awareness survey of specialists in the outpatient sector and their patients, covering risk factors and relevant aspects of prevention, diagnosis, and treatment of osteoporosis in Switzerland. We highlight commonalities and differences across regions and medical fields in order to portray the current state of health care and identify relevant issues that could become part of a future common approach to tackling osteoporosis.

Our survey involved specialists from four medical fields (general practice, rheumatology, endocrinology, and gynecology) that are likely to encounter patients in need of osteoporosis prevention or treatment, and a substantial number of physicians and their patients participated in the survey. Both factors set a good foundation for the collection of representative field data on osteoporosis. However, certain limitations apply regarding the survey sample. Proportion of rheumatologists was higher compared to the estimated value for the Swiss outpatient sector [24], possibly facilitated by selection bias stemming from the composition of the potential contacts’ list used for recruitment of participating physicians. The Italian-speaking region was not represented in the subpopulation of gynecologists or endocrinologists, leading to certain level of information bias. Together with a comparatively lower number of participants from that region, this could have for example influenced our finding of the Italian-speaking region having the highest prevalence of fragility fractures relative to the respective number of participants. Patient population was predominantly female and on average somewhat older than the population of Switzerland, which also represents a source of information bias [26]. Limitations generally associated with survey research such as recall bias also apply [27]. Furthermore, reporting bias can occur, as respondents’ knowledge and perspective might impact individual responses. For instance, different prevalence of various steps taken after a fragility fracture found in the accounts of patients and physicians is likely at least partly attributable to their correct identification and recollection. However, multiple relevant criteria for gathering and analyzing representative field data were fulfilled, illustrating strengths of the survey. Regional distribution of the overall survey sample corresponds well to the language distribution in Switzerland, speaking to the representative nature of our national survey [25]. Patient population includes both genders and spans across different age groups, representative of different needs regarding osteoporosis prevention and treatment. Particularly vulnerable patient populations with underlying medical conditions or treatments potentially increasing the risk of osteoporosis occurrence, as well as patients with manifested osteoporosis involving fragility fractures and targeted medical treatment were also represented in the survey. We therefore remain confident that our results provide relevant novel insights into the awareness and management of osteoporosis.

Results of our survey demonstrate the presence of various risk factors for the occurrence of osteoporosis. Female gender and advanced age are established risk factors in this regard, and a large treatment gap has previously been described in routine primary care across Europe for women aged ≥70 years with an assessed increased risk of fragility fractures [1,6,28]. Our survey additionally uncovers the extent of the presence of various other risk factors concerning patients’ medical history or nutrition and lifestyle. Relevance of underlying medical conditions and/or treatments was apparent both in terms of their presence in the overall survey population, affected to almost one third, as well as in the context of particular subgroups. For instance, inflammatory rheumatic disease and glucocorticoid treatment, also included as clinical risk factors in the FRAX assessment tool [8], were most present in rheumatology patients, suggesting the importance of an individualized approach towards mitigating the risk of osteoporosis in patients of different specialists.

Although only a small minority of patients followed a particular diet, the intake of dairy products fell short of seven portions per week. Low consumption of dairy products is consistent with previous assessment of nutrition in Switzerland [29]. Furthermore, observed regional differences in the intake of milk and fish also support previous findings [30]. Although it is conceivable that sufficient calcium supply can still be achieved through an adequate combination of appropriate foods, our results nevertheless suggest caution is warranted in ensuring optimal supply through nutrition or, if required, supplements. Based on our findings, this might be particularly relevant for endocrinology patients, since they were somewhat more frequently on a diet compared to other patient groups. Although a majority of patients in our survey engaged in weekly exercise, its frequency was often below the recommendations for preventing and counteracting osteoporosis [31]. This issue might be tackled by systematic screening and counselling of insufficiently active patients, which has previously proved feasible in general practice [32]. Other risk factors, such as family history of hip fractures, smoking or elevated alcohol intake [8], were expectedly also present with patients of different specialists, highlighting the importance of their awareness across different medical fields.

Calcium and vitamin D supplements are likely a highly cost-effective measure whose wider use could considerably reduce fractures and related costs [13]. However, our results indicate that the majority of patients did not take supplements, which is especially notable in view of the previously reported shortfall in upholding the vitamin D recommendations in most European countries [33]. Out of the three supplement variants covered by our survey, intake of calcium was the least represented. Although this form of supplementation has been questioned in the context of fracture prevention [34,35], a cross-sectional study on the use of vitamin or dietary supplements in Lausanne, Switzerland, previously found calcium to be the third most used supplement, with reported 6.6% of consumers [36]. Our present findings in patients of various specialists correlate well with this previous result. It is nevertheless important to note that nutritional sources are primarily recommended for calcium intake and that calcium supplements combined with adequate vitamin D replacement should be reserved for patients with inadequate dietary intake of calcium [35]. As high prevalence of hypovitaminosis D had previously been established in a Swiss rheumatology outpatient population [37], our observation that approximately 2/5 of rheumatology patients use vitamin D alone or combined with calcium suggests there might be potential for further improvement.

Our results indicate osteoporosis is relevant for the physicians’ daily practice, admittedly with some differences between various specialists. Although our findings show that various risk factors for osteoporosis are present among surveyed patients and that patient initiative is relevant for introducing diagnostic measures, their rather low awareness of the chronic nature of osteoporosis still suggests a substantial knowledge gap regarding the disease. Advancing patient education, as recommended by various guidelines [2,14], as well as communication between physicians and patients could help to bridge the gap and accomplish more consistent, targeted testing for osteoporosis. Confidence in treating osteoporosis and feeling adequately trained for such treatment varied among physicians, suggesting a need for more targeted training for certain specialists. This notion is supported by a previous finding of insufficient education on bone healthcare impacting the awareness within the gynecological field in Switzerland [38]. In addition, our results demonstrate that the approach undertaken by the physicians in vital aspects of prevention, diagnosis and treatment differs across regions and areas of expertise, illustrating the need for more standardization of care on a national level and across disciplines.

## 5. Conclusions

Although certain regional challenges can require regional solutions, our findings suggest more patient education as well as cross-regional dialog among physicians might be beneficial for raising awareness on osteoporosis, identifying potential areas of improvement, and facilitating standardization of care across Switzerland. With this approach, management of osteoporosis could be improved through different stakeholders of the health care system.

## Figures and Tables

**Table 1:** Distribution of surveyed physicians and patients compared to statistical data for Switzerland.

Proportion of Various Specialists (%)			
Specialty	Switzerland FMH *	BHA Survey	
GPs	80.4	64.9	
Rheumatologists	1.5	16.8	
Gynecologists	14.5	12.2	
Endocrinologists	3.7	6.1	
**Regional distribution of survey sample vs. language distribution in Switzerland (BFS 2017, %)**			
Region/language	Switzerland	Survey physicians (*n* = 262)	Survey patients (*n* = 9065)
German	62.6	61.5	64.2
Italian	8.2	4.6	4.2
French	22.9	34.0	31.6

* Calculation performed based on FMH data on the number of physicians belonging to the four listed target groups of the survey, recorded as employed in the Swiss outpatient sector in 2018.

**Table 2:** Family history of hip fractures.

Has Any of Your Parents or Siblings Suffered a Hip Fracture?	Percentage of Patients								
	All Patients	Patients with Fragility Fractures	Patients of Different Physicians				Patients of Different Regions		
			GPs	Rheumatologists	Gynecologists	Endocrinologists	German	Italian	French
Yes.	8.1	11.7	8.1	10.2	4.7	11.1	7.9	12.9	8.1
No.	78.6	75.1	77.7	77.0	85.6	76.6	77.8	73.1	80.9
I do not know.	8.1	11.4	8.8	8.2	4.9	6.9	8.9	6.3	6.7
Not specified.	5.2	1.9	5.4	4.7	4.8	5.4	5.5	7.7	4.4

**Table 3:** Intake of calcium/vitamin D supplements among patients of different physicians.

Supplement Intake	GP Patients (%)	Rheumatology Patients (%)	Gynecology Patients (%)	Endocrinology Patients (%)
Not specified	3.1	2.7	3.4	1.9
No calcium or vitamin D intake	56.1	45	57.3	43.3
Calcium supplementation	5.6	6	4.2	5.9
Vitamin D supplementation	18.5	20.2	19.9	25.1
Calcium + vitamin D supplementation	18	28	15.9	25.3

**Table 4:** Evaluation and treatment of fragility fractures as reported by surveyed patients and physicians.

Reported Evaluation and Treatment of Fragility Fractures	Patients (%)	Physicians (%)
X-ray	62.8	47
Bone density measurement (DXA)	37.5	88.6
Referral to a specialist	33.8	29.4
Treatment (supplements, medicines)	20.8	Prescription: Calcium 25.2 Vitamin D: 32.1 Calcium + Vitamin D: 70.2
Use of risk assessment tool (e.g., questionnaire or FRAX)	5.4	55.3
None of the listed steps	10.4	0.8

## Supplementary Materials

The following supporting information can be downloaded at: https://www.mdpi.com/article/10.3390/healthcare10020295/s1, S1: BHAS patient questionnaire (German), S2: BHAS patient questionnaire (Italian), S3: BHAS patient questionnaire (French), S4: BHAS physician questionnaire (German), S5: BHAS physician questionnaire (Italian), S6: BHAS physician questionnaire (French).
