# Supplementary material for: Risk Factors, Manifestation, and Awareness of Osteoporosis among Patients of Various Specialists in Switzerland: Results of a National Survey
Source: Healthcare (Basel). 2022 Feb 3;10(2):295. doi: 10.3390/healthcare10020295 (PMC8871550; doi:10.3390/healthcare10020295)
Supplement: Supplementary file 1 [file healthcare-10-00295-s001.zip › S1.pdf]

PATIENTEN-FRAGEBOGEN ZUR KNOCHENGESUNDHEIT IN DER SCHWEIZ

Sehr geehrte Patientin, sehr geehrter Patient, vielen Dank, dass Sie uns bei unserer Umfrage zur Knochengesundheit in der Schweiz unterstützen! Beantworten Sie bitte folgende Fragen:

1) Geschlecht: männlich ☐ weiblich ☐ **2) Geburtsdatum:** ____________________

3) Körpergrösse: ________ cm **4) Körpergewicht:** ________ kg

5) Ist Osteoporose eine chronische Erkrankung?
Ja ☐ Nein ☐ Ich weiss es nicht ☐

Beantworten Sie bitte folgende Fragen bezüglich Ihrer Ernährungs- / Lebensweise:
6) Wie viele Portionen dieser Lebensmittel (entsprechend 100 g / 1 dl) nehmen Sie pro Woche zu sich?

	weniger als 7 Portionen	mehr als 7 Portionen
**Käse** [a]	☐	☐
**Milch** [b]	☐	☐
**Wasser** [b]	☐	☐
**Cerealien** [c]	☐	☐
**Joghurt** [d]	☐	☐
**Fleisch** [e]	☐	☐
**Fisch** [f]	☐	☐
**Grünes Gemüse** [g]	☐	☐

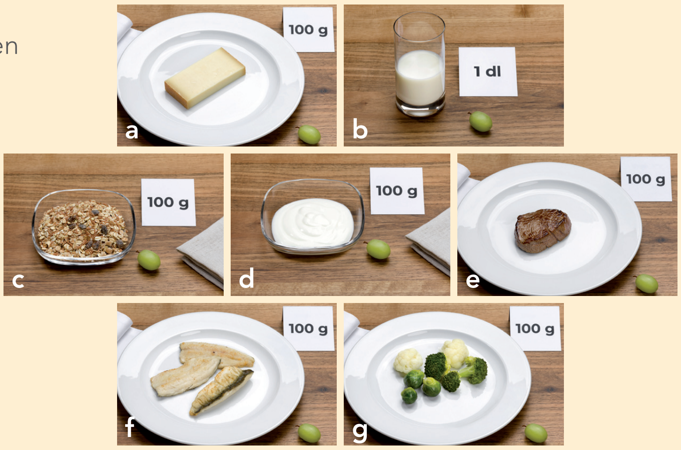

7) Halten Sie eine bestimmte Ernährungsweise / Diät ein?
Vegan ☐ Vegetarisch ☐ Andere ☐ Keine Diät ☐

8) Nehmen Sie Calcium- und / oder Vitamin D-Nahrungsergänzungsmittel ein?
Wenn Ihre Antwort „nein" lautet, bitte weiter mit Frage 10.
Nein ☐ Calcium ☐ Vitamin D ☐ Calcium + Vitamin D ☐

9) Wenn ja, wie oft oder regelmässig nehmen Sie Calcium / Vitamin D ein?
täglich ☐ wöchentlich ☐ nur im Winter ☐ unregelmässig ☐

10) Wie oft treiben Sie Sport?
(Wöchentliche sportliche Betätigung; 30 Minuten je Einheit)
1 bis 2 x ☐ 3 bis 4 x ☐ 5 bis 7 x ☐ mehr als 7 x ☐ nie ☐

11) Rauchen Sie?
Ja ☐ Nein ☐

12) Nehmen Sie täglich mehr als 8–10 g Alkohol zu sich (entspricht einem Glas Bier = 300 ml / 3 dl oder einem Glas Wein = 100 ml / 1 dl)?
Ja ☐ Nein ☐

KAL.0716.055.5.0119.300

Sie können unerwünschte Arzneimittelwirkungen, die bei einem Produkt von Mylan Pharma GmbH / MEDA Pharma GmbH / MEDA Pharmaceuticals Switzerland GmbH auftreten, an unser Pharmakovigilanz-Team unter pv.switzerland@mylan.com, Fax: 041 768 48 11 melden.

13) Nehmen Sie folgende Medikamente ein?
(Mehrfachantworten möglich)

- ☐ Glukokortikoide zum Einnehmen über mehr als 3 Monate (z. B. Kortison, Prednison)
- ☐ Antidepressiva
- ☐ Anti-Hormontherapie bei Brustkrebs oder Prostatakrebs
- ☐ Säureblocker
- ☐ Keines der genannten

14) Wurden bei Ihnen folgende Krankheiten diagnostiziert bzw. Eingriffe vorgenommen?
(Mehrfachantworten möglich)

- ☐ Entzündliche rheumatische Erkrankung
- ☐ Zöliakie oder „Sprue"
- ☐ Magenbypass
- ☐ Überfunktion der Nebenschilddrüse
- ☐ HIV
- ☐ Diabetes
- ☐ Chronische entzündliche Darmerkrankung (z. B. Morbus Crohn, Colitis ulcerosa)
- ☐ Keine der genannten

15) Erhalten Sie eine Behandlung gegen Osteoporose?
Ja ☐ Nein ☐

Fragen nur für Patientinnen:
16) Sind Sie in den Wechseljahren (Menopause)?
Ja ☐ Nein ☐ Ich weiss es nicht ☐

17) Erhalten Sie eine Hormonersatztherapie?
Ja ☐ Nein ☐

Beantworten Sie bitte folgende Fragen bezüglich Ihrer Knochengesundheit:
18) Machen Sie sich Sorgen, dass Ihre Knochen leicht brechen könnten?
Ja ☐ Nein ☐

19) Hatten Sie schon einmal einen Knochenbruch ohne äussere Einwirkungen?
(Beispiele für äussere Einwirkungen: Sturz, Unfall o. ä.)
Wenn Ihre Antwort „nein" lautet, bitte weiter mit Frage 23.
Ja ☐ Nein ☐

20) Wenn ja, in welchem Alter: ________ Jahre

21) Welcher Knochen war gebrochen?
(Mehrfachantworten möglich)

- ☐ Hüfte
- ☐ Wirbelsäule
- ☐ Handgelenk
- ☐ Sonstiger

22) Was ist nach dem Knochenbruch geschehen?
(Mehrfachantworten möglich)
☐ Beurteilung des Frakturrisikos (z. B. mittels Fragebogen)
☐ Knochendichtemessung
☐ Behandlung (Nahrungsergänzungsmittel, bestimmte Medikamente)
☐ Röntgenaufnahmen
☐ Überweisung an einen Spezialisten
☐ Keine der genannten Massnahmen

23) Sind Sie beim Gehen unsicher oder haben Sie Angst vor Stürzen?
Ja ☐ Nein ☐

24) Hatte eines Ihrer Elternteile oder Ihre Geschwister eine Hüftfraktur?
Ja ☐ Nein ☐ Ich weiss es nicht ☐

25) Hat Ihr Arzt Ihnen schon einmal ein Medikament verordnet?
Wenn Ihre Antwort „nein" lautet, bitte weiter mit Frage 27.
Ja ☐ Nein ☐

26) Haben Sie dieses nach Anweisung Ihres Arztes eingenommen?
Immer ☐ Oft ☐ Selten ☐ Nie ☐

27) Hat Ihr Arzt Ihnen Nahrungsergänzungsmittel verordnet (z. B ein Vitaminpräparat, Magnesium, Calcium etc.)?
Wenn Ihre Antwort „nein" lautet, bitte weiter mit Frage 30.
Ja ☐ Nein ☐

28) Haben Sie diese nach Anweisung Ihres Arztes eingenommen?
Immer ☐ Oft ☐ Selten ☐ Nie ☐

29) Ihr Arzt verordnet Ihnen Medikamente / Nahrungsergänzungsmittel und Sie nehmen diese nach Anweisung ein. Was sind die Gründe für die Einnahme? *(Mehrfachnennung möglich)*

**Ich vertraue meinem Arzt**	Medikamente ☐	Nahrungsergänzungsmittel ☐
**Linderung der Symptome**	Medikamente ☐	Nahrungsergänzungsmittel ☐

30) Ihr Arzt verordnet Ihnen Medikamente / Ergänzungsmittel und Sie nehmen diese nicht ein. Was sind die Gründe für die Nicht-Einnahme? *(Mehrfachnennung möglich)*
☐ Weil es Chemie ist
☐ Bin nicht überzeugt, dass es mir hilft
☐ Keine Symptome / keine Notwendigkeit
☐ Allgemeine Bedenken über mögliche Nebenwirkungen
☐ Ich nehme alternative Medikamente (Komplementärmedizin)

31) Sind Sie der Meinung, dass es sich bei Calcium- / Vitamin D-Nahrungsergänzungsmitteln um Lifestyle-Mittel / Modeerscheinung handelt?
Ja ☐ Nein ☐

Sie können unerwünschte Arzneimittelwirkungen, die bei einem Produkt von Mylan Pharma GmbH / MEDA Pharma GmbH / MEDA Pharmaceuticals Switzerland GmbH auftreten, an unser Pharmakovigilanz-Team unter pv.switzerland@mylan.com, Fax: 041 768 48 11 melden.
