# Supplementary material for: Risk Factors, Manifestation, and Awareness of Osteoporosis among Patients of Various Specialists in Switzerland: Results of a National Survey
Source: Healthcare (Basel). 2022 Feb 3;10(2):295. doi: 10.3390/healthcare10020295 (PMC8871550; doi:10.3390/healthcare10020295)
Supplement: Supplementary file 1 [file healthcare-10-00295-s001.zip › S2.pdf]

QUESTIONARIO PAZIENTI PER LA SALUTE DELLE OSSA IN SVIZZERA

Gentile paziente, la ringraziamo per il tempo dedicato al nostro sondaggio relativo alla salute delle ossa in Svizzera! La preghiamo di rispondere alle seguenti domande:

1) Sesso: maschile ☐ femminile ☐ **2) Data di nascita:** ____________________

3) Statura: ________ cm **4) Peso:** ________ kg

5) L'ostéoporose est-elle une maladie chronique?
Sì ☐ No ☐ Non so ☐

La preghiamo di rispondere alle seguenti domande sulle sue abitudini alimentari:
6) Quante porzioni assume in una settimana di questo alimento (una porzione = 100 g / 1 dl)?

	meno di 7 porzioni	più di 7 porzioni
**formaggio** [a]	☐	☐
**latte** [b]	☐	☐
**acqua** [b]	☐	☐
**cereali** [c]	☐	☐
**yogurt** [d]	☐	☐
**carne** [e]	☐	☐
**pesce** [f]	☐	☐
**verdura** [g]	☐	☐

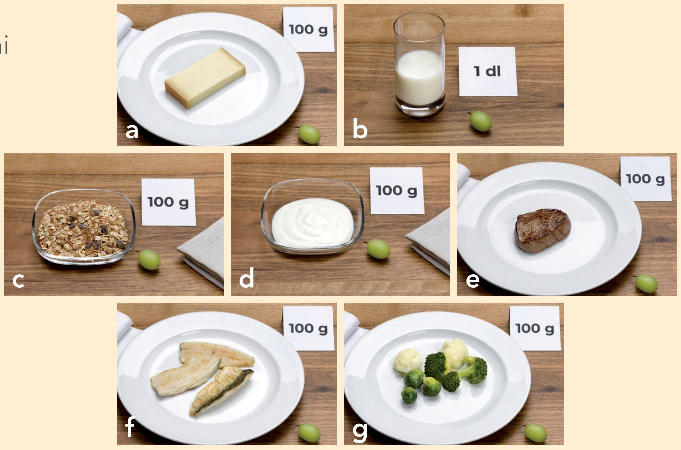

7) Segue un tipo di alimentazione / dieta particolare?
Vegana ☐ Vegetariana ☐ Altro ☐ Nessuna dieta ☐

8) Assume integratori alimentari per il calcio e / o la vitamina D?
Se la sua risposta è "no", prosegua con la domanda 10.
No ☐ Calcio ☐ Vitamina D ☐ Calcio + Vitamina D ☐

9) Se la risposta è sì, con che frequenza o regolarità assume calcio / vitamina D?
tutti i giorni ☐ tutte le settimane ☐ solo in inverno ☐ irregolarmente ☐

10) Con che frequenza pratica uno sport?
(Attività sportiva settimanale; 30 minuti per sessione)
1-2 volte ☐ 3-4 volte ☐ 5-7 volte ☐ più di 7 volte ☐ mai ☐

11) Fuma?
Si ☐ No ☐

12) Assume ogni giorno più di 8-10 g di alcol (corrispondenti a un bicchiere di birra = 300 ml / 3 dl o un bicchiere di vino = 100 ml / 1 dl)??
Si ☐ No ☐

KAL.0716.055.5.0119.300

Può riferire gli effetti indesiderati che si verificano con un prodotto di Mylan Pharma GmbH / MEDA Pharma GmbH / MEDA Pharmaceuticals Switzerland GmbH al nostro team Farmacovigilanza all'indirizzo pv.switzerland@mylan.com, Fax: 041 768 48 11.

13) Assume i seguenti medicamenti?
(più risposte sono possibili)

- ☐ Glucocorticoidi da assumere per più di 3 mesi (ad es. cortisone, prednisone)
- ☐ Antidepressivi
- ☐ Terapia antiormonale per cancro della mammella o della prostata
- ☐ Antiacidi
- ☐ Nessuno di questi

14) Le sono state diagnosticate le seguenti malattie o sono stati eseguiti i seguenti interventi?
(più risposte sono possibili)

- ☐ Malattia reumatica infiammatoria
- ☐ Celiachia o "sprue"
- ☐ Bypass gastrico
- ☐ Iperattività paratiroidea
- ☐ HIV
- ☐ Diabete
- ☐ Infiammazione intestinale cronica (ad es. morbo di Crohn, colite ulcerosa)
- ☐ Nessuno di questi

15) Pratica un trattamento contro l'osteoporosi?
Si ☐ No ☐

Domande solo per le pazienti:
16) È in menopausa?
Si ☐ No ☐ Non so ☐

17) Pratica una terapia ormonale sostitutiva?
Si ☐ No ☐

La preghiamo di rispondere alle seguenti domande sulla salute delle sue ossa:
18) È preoccupata che le sue ossa possano fratturarsi facilmente?
Si ☐ No ☐

19) Ha già avuto una frattura ossea senza effetti esterni?
(Esempi di effetti esterni: caduta, incidente e simili)
Se la sua risposta è "no", prosegua con la domanda 23.
Si ☐ No ☐

20) Se sì, a che età? ________ Anni

21) Quali ossa si è fratturata?
(più risposte sono possibili)

- ☐ Anca
- ☐ Colonna vertebrale
- ☐ Polso
- ☐ Altro

22) Che cosa è successo dopo la frattura?
(più risposte sono possibili)
☐ Valutazione del rischio di fratture (ad es. mediante questionario)
☐ Misurazione della densità ossea
☐ Trattamento (integratori alimentari, determinati medicamenti)
☐ Radiografie
☐ Invio da uno specialista
☐ Nessuno di questi provvedimenti

23) Si sente insicura quando cammina o ha paura di cadere?
Si ☐ No ☐

24) Uno dei suoi genitori, fratelli o sorelle ha mai avuto una frattura dell'anca?
Si ☐ No ☐ Non so ☐

25) Il suo medico le ha già prescritto un medicamento in passato?
Se la sua risposta è "no", prosegua con la domanda 27.
Si ☐ No ☐

26) Lo ha preso secondo le istruzioni del suo medico?
Sempre ☐ Spesso ☐ Raramente ☐ Mai ☐

27) Il suo medico le ha prescritto integratori alimentari (ad es. preparato vitaminico, magnesio, calcio ecc.)
Se la sua risposta è "no", prosegua con la domanda 30.
Si ☐ No ☐

28) Li ha presi secondo le istruzioni del suo medico?
Sempre ☐ Spesso ☐ Raramente ☐ Mai ☐

29)) Il suo medico le prescrive medicamenti / integratori alimentari e lei li assume secondo le istruzioni. Per quale motivo? *(più risposte sono possibili)*

**Mi fido del mio medico**	Medicamenti ☐	Integratori alimentari ☐
**Riduzione dei sintomi**	Medicamenti ☐	Integratori alimentari ☐

30) Il suo medico le prescrive medicamenti / integratori e lei non li assume. Per quale motivo?
(più risposte sono possibili)
☐ Perché si tratta di roba chimica
☐ Non sono convinta che serva
☐ Nessun sintomo / nessun bisogno
☐ Preoccupazione in generale di possibili effetti indesiderati
☐ Prendo medicamenti alternativi (medicina complementare

31) Ritiene che gli integratori alimentari a base di calcio / vitamina D siano un fatto di lifestyle / di moda?
Si ☐ No ☐

Può riferire gli effetti indesiderati che si verificano con un prodotto di Mylan Pharma GmbH / MEDA Pharma GmbH / MEDA Pharmaceuticals Switzerland GmbH al nostro team Farmacovigilanza all'indirizzo pv.switzerland@mylan.com, Fax: 041 768 48 11.
