# Supplementary material for: Risk Factors, Manifestation, and Awareness of Osteoporosis among Patients of Various Specialists in Switzerland: Results of a National Survey
Source: Healthcare (Basel). 2022 Feb 3;10(2):295. doi: 10.3390/healthcare10020295 (PMC8871550; doi:10.3390/healthcare10020295)
Supplement: Supplementary file 1 [file healthcare-10-00295-s001.zip › S3.pdf]

QUESTIONNAIRE SUR LA SANTÉ OSSEUSE EN SUISSE

Chère patiente, cher patient, merci beaucoup pour votre participation à notre enquête sur la santé osseuse en Suisse! Veuillez répondre aux questions suivantes:

1) Sexe: masculin ☐ féminin ☐ **2) Date de naissance:** ____________

3) Taille: ________ cm **4) Poids:** ________ kg

5) L'ostéoporose est-elle une maladie chronique?
Oui ☐ Non ☐ Je ne sais pas ☐

Veuillez répondre aux questions suivantes concernant votre alimentation / mode de vie:
6) Combien de portions de ces produits (correspondant à 100 g / 1 dl) consommez-vous chaque semaine?

	moins de 7 portions	plus de 7 portions
**Fromage** [a]	☐	☐
**Lait** [b]	☐	☐
**Eau** [b]	☐	☐
**Céréales** [c]	☐	☐
**Yaourt** [d]	☐	☐
**Viande** [e]	☐	☐
**Poisson** [f]	☐	☐
**Légumes verts** [g]	☐	☐

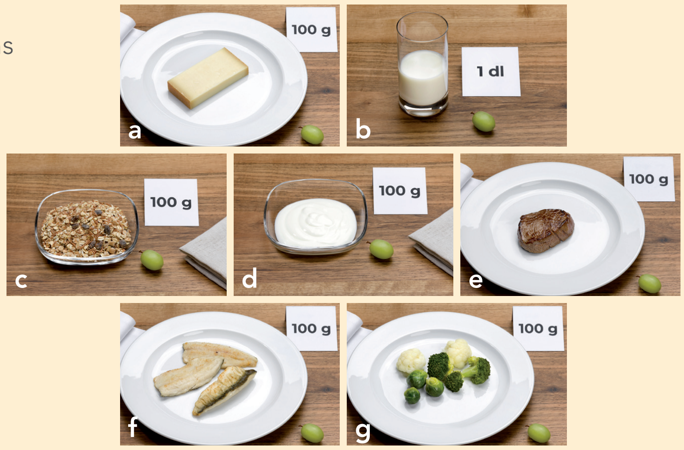

7) Suivez-vous un régime particulier?
Végane ☐ Végétarien ☐ Autre ☐ Aucun régime ☐

8) Prenez-vous des compléments de calcium et / ou de vitamine D?
Si votre réponse est « non », veuillez passer à la question 10.
Non ☐ Calcium ☐ Vitamine D ☐ Calcium et vitamine D ☐

9) Si oui, à quelle fréquence prenez-vous du calcium / de la vitamine D?
tous les jours ☐ chaque semaine ☐ seulement pendant l'hiver ☐ de façon irrégulière ☐

10) À quelle fréquence pratiquez-vous une activité physique?
(Nombre de séances par semaine ; 30 min par séance)
1-2 fois ☐ 3-4 fois ☐ 5-7 fois ☐ plus de 7 fois ☐ jamais ☐

11) Fumez-vous?
Oui ☐ Non ☐

12) Buvez-vous plus de 8-10 g d'alcool par jour (équivaut à un verre de bière = 3dl ou de vin = 1dl)?
Oui ☐ Non ☐

KAL.0716.055.5.0119.300

Vous pouvez signaler les réactions indésirables à un produit Mylan Pharma GmbH / MEDA Pharma GmbH / MEDA Pharmaceuticals Switzerland GmbH auprès de notre équipe de Pharmacovigilance par e-mail à pv.switzerland@mylan.com Fax: 041 768 48 11.

13) Prenez-vous l'un des traitements suivants?
(Plusieurs réponses possibles)
- ☐ Glucocorticoïdes oraux pris pendant plus de 3 mois (cortisone, prednisone, par exemple)
- ☐ Antidépresseurs
- ☐ Traitement anti-hormonal dans le cadre d'un cancer du sein ou de la prostate
- ☐ Anti-acides
- ☐ Aucune des propositions

14) L'une des maladies ou intervention suivantes a-t-elle été diagnostiquée ou eu lieu chez vous?
(Plusieurs réponses possibles)
- ☐ Maladies rhumatismales inflammatoires
- ☐ Maladie cœliaque ou « sprue »
- ☐ Bypass gastrique
- ☐ Hyperparathyroïdie
- ☐ Infection VIH
- ☐ Diabète
- ☐ Maladie inflammatoire chronique de l'intestin (maladie de Crohn, rectocolite hémorragique, p. ex.)
- ☐ Aucune des propositions

15) Suivez-vous un traitement contre l'ostéoporose?
Oui ☐ Non ☐

Questions destinées uniquement aux patientes de sexe féminin:
16) Êtes-vous ménopausée?
Oui ☐ Non ☐ Je ne sais pas ☐

17) Prenez-vous un traitement hormonal substitutif?
Oui ☐ Non ☐

Veuillez répondre aux questions suivantes concernant votre santé osseuse:
18) Vous sentez-vous préoccupé(e) par votre fragilité osseuse?
Oui ☐ Non ☐

19) Avez-vous déjà eu une fracture osseuse sans influence extérieure?
Influences extérieures sont p. ex. chute, accident, etc.
Si votre réponse est « non », veuillez passer à la question 23.
Oui ☐ Non ☐

20) Si oui, à quel âge: ________ ans

21) Quel os a été touché par la fracture?
(Plusieurs réponses possibles)
- ☐ Hanche
- ☐ Colonne
- ☐ Poignet
- ☐ Autre

22) Que s'est-il passé ensuite?
(Plusieurs réponses possibles)
☐ Évaluation du risque de fracture (p. ex. à l'aide d'un questionnaire)
☐ Mesure de la densité osseuse
☐ Traitement (compléments, traitement spécifique)
☐ Radiographies
☐ Renvoi vers un spécialiste
☐ Aucune des propositions

23) Êtes-vous peu assuré(e) quand vous marchez ou avez-vous peur de tomber?
Oui ☐ Non ☐

24) L'un de vos parents ou frères et sœurs a-t-il eu une fracture de la hanche?
Oui ☐ Non ☐ Je ne sais pas ☐

25) Votre médecin vous a-t-il prescrit un traitement?
Si votre réponse est « non », veuillez passer à la question 27.
Oui ☐ Non ☐

26) Prenez-vous ce traitement?
Toujours ☐ Souvent ☐ Rarement ☐ Jamais ☐

27) Votre médecin vous a-t-il prescrit des compléments?
(p. ex. vitamines, magnésium, calcium, etc.)
Si votre réponse est « non », veuillez passer à la question 30.
Oui ☐ Non ☐

28) Prenez-vous ces compléments?
Toujours ☐ Souvent ☐ Rarement ☐ Jamais ☐

29) Votre médecin vous prescrit un traitement/complément et vous le prenez. Pour quelles raisons le prenez-vous? *(Plusieurs réponses possibles)*

**Je fais confiance à mon médecin**	traitement ☐	compléments ☐
**Pour réduire mes symptômes**	traitement ☐	compléments ☐

30) Votre médecin vous prescrit un traitement / complément et vous ne le prenez pas. Pour quelles raisons ne le prenez-vous pas? *(Plusieurs réponses possibles)*
☐ Parce que c'est chimique
☐ Pas convaincu(e) que cela m'aide
☐ Pas de symptômes/pas besoin
☐ Inquiétudes générales concernant les effets indésirables éventuels
☐ Je prends un traitement alternatif (médecine douce)

31) Pensez-vous que le calcium / la vitamine D sont des produits « mode de vie »?
Oui ☐ Non ☐

Vous pouvez signaler les réactions indésirables à un produit Mylan Pharma GmbH / MEDA Pharma GmbH / MEDA Pharmaceuticals Switzerland GmbH auprès de notre équipe de Pharmacovigilance par e-mail à pv.switzerland@mylan.com Fax: 041 768 48 11.
