# Supplementary material for: Risk Factors, Manifestation, and Awareness of Osteoporosis among Patients of Various Specialists in Switzerland: Results of a National Survey
Source: Healthcare (Basel). 2022 Feb 3;10(2):295. doi: 10.3390/healthcare10020295 (PMC8871550; doi:10.3390/healthcare10020295)
Supplement: Supplementary file 1 [file healthcare-10-00295-s001.zip › S4.pdf]

ARZT-FRAGEBOGEN ZUR KNOCHENGESUNDHEIT IN DER SCHWEIZ

Sehr geehrte Ärztin, sehr geehrter Arzt, vielen Dank, dass Sie uns bei unserer Umfrage zur Knochengesundheit in der Schweiz unterstützen! Beantworten Sie bitte folgende Fragen:

1) Informationen zum Fachgebiet

- ☐ Allgemeinmedizin
- ☐ Rheumatologie
- ☐ Gynäkologie
- ☐ Orthopädie
- ☐ Endokrinologie
- ☐ Anderes Fachgebiet

2) Wie viele Patienten mit Osteoporose behandeln Sie (ausgehend von der Gesamtheit Ihrer Patienten)?

☐ Keine ☐ < 20 % ☐ 20–50 % ☐ > 50 %

3) Wie gehen Sie als nächstes vor, wenn bei Ihnen ein Patient mit einer atraumatischen Fraktur vorstellig wird?

(Mehrfachantworten möglich)

- ☐ Keine weitere Massnahme
- ☐ Einsatz von Beurteilungsinstrumenten zum Frakturrisiko (z. B. FRAX, TOP-TOOL)
- ☐ Knochendichtemessung
- ☐ Röntgen
- ☐ Überweisung an einen Spezialisten
- ☐ Verordnung eines Calcium-Nahrungsergänzungsmittels
- ☐ Verordnung eines Vitamin D-Nahrungsergänzungsmittels
- ☐ Verordnung eines kombinierten Calcium- / Vitamin D-Nahrungsergänzungsmittels
- ☐ Befragung zur Ernährungs- und Lebensweise

4) Was sind die Gründe dafür, dass Sie Patienten mit Osteoporose behandeln bzw. nicht behandeln?

(Mehrfachantworten möglich)

- ☐ Ich fühle mich bei der Behandlung dieser Patienten sicher
- ☐ Ich möchte die Gesundheit der Patienten erhalten
- ☐ Ich bin für die Behandlung von Osteoporose nicht ausgebildet
- ☐ Ich überweise die Patienten an Spezialisten
- ☐ Ich bin der Meinung, dass es zu teuer wäre, diese Patienten selbst zu behandeln

5) Welchen Stellenwert haben die folgenden Krankheiten in Ihrer täglichen Praxis?

**Diabetes**	wichtig	0	1	2	3	4	5	nicht wichtig
**Osteoporose**	wichtig	0	1	2	3	4	5	nicht wichtig

6) Auf Grundlage welcher Kriterien führen Sie eine Untersuchung auf Osteoporose durch?

(Mehrfachantworten möglich)

- ☐ Klinische Kriterien
- ☐ Auf Initiative des Patienten
- ☐ Screening (z. B. FRAX, TOP-TOOL)
- ☐ Mangelernährung

7) Wann verordnen Sie ein Calcium- / Vitamin D-Nahrungsergänzungsmittel?

(Mehrfachantworten möglich)

- ☐ Bei Mangelernährung
- ☐ Als ergänzende Behandlung
- ☐ Mangelnde Calcium-/Vitamin D-Versorgung
- ☐ Bauchgefühl
- ☐ Nie

Sie können unerwünschte Arzneimittelwirkungen, die bei einem Produkt von Mylan Pharma GmbH / MEDA Pharma GmbH / MEDA Pharmaceuticals Switzerland GmbH auftreten, an unser Pharmakovigilanz-Team unter pv.switzerland@mylan.com, Fax: 041 768 48 11 melden. Dies ersetzt jedoch nicht die Meldepflicht gegenüber der Behörde gemäss HMG.

KAL.0716.055.5.0119.300
