# Supplementary material for: Risk Factors, Manifestation, and Awareness of Osteoporosis among Patients of Various Specialists in Switzerland: Results of a National Survey
Source: Healthcare (Basel). 2022 Feb 3;10(2):295. doi: 10.3390/healthcare10020295 (PMC8871550; doi:10.3390/healthcare10020295)
Supplement: Supplementary file 1 [file healthcare-10-00295-s001.zip › S5.pdf]

QUESTIONARIO PER I MEDICI PER LA SALUTE DELLE OSSA IN SVIZZERA

Gentile Dottoressa, Gentile Dottore, la ringraziamo per il suo supporto al nostro sondaggio relativo alla salute delle ossa in Svizzera! La preghiamo di rispondere alle seguenti domande:

1) Informazioni sulla specializzazione

☐ Medicina generale ☐ Reumatologia ☐ Ginecologia
☐ Orthopedia ☐ Endocrinologia ☐ Altra specializzazione

2) Quanti pazienti con osteoporosi tratta (sul totale dei suoi pazienti)?

☐ Nessuno ☐ < 20 % ☐ 20–50 % ☐ > 50 %

3) Come procede quando le si presenta un paziente con una frattura atraumatica?
(più risposte sono possibili)

☐ Nessun provvedimento ulteriore
☐ Impiego di strumenti di valutazione del rischio di frattura (ad es. FRAX, TOP-TOOL)
☐ Misurazione della densità ossea
☐ Radiografie
☐ Invio da uno specialista
☐ Prescrizione di un integratore alimentare per il calcio
☐ Prescrizione di un integratore alimentare per la vitamina D
☐ Prescrizione di un integratore alimentare combinato per calcio / vitamina D
☐ Domande sulle abitudini alimentari e di comportamento

4) Quali sono i motivi per cui tratta o non tratta pazienti con osteoporosi?
(più risposte sono possibili)

☐ Mi sento sicuro nel trattamento di questi pazienti
☐ Vorrei mantenere in salute i pazienti
☐ Non ho ricevuto una formazione per il trattamento dell'osteoporosi
☐ Mando i pazienti dagli specialisti
☐ Sono del parere che costerebbe troppo trattare in proprio questi pazienti

5) Quale ordine d'importanza hanno le seguenti malattie nella sua attività quotidiana?

**Diabete**	importante	0	1	2	3	4	5	non importante
**Osteoporosi**	importante	0	1	2	3	4	5	non importante

6) Sulla base di quali criteri esegue un esame per l'osteoporosi?
(più risposte sono possibili)

☐ Criteri clinici ☐ Su iniziativa del paziente
☐ Screening (ad es. FRAX, TOP-TOOL) ☐ Malnutrizione

7) Quando prescrive un integratore alimentare per calcio / vitamina D?
(più risposte sono possibili)

☐ In caso di malnutrizione ☐ Come trattamento integrativo
☐ Carenza di apporto di calcio / vitamina D ☐ Istinto ☐ Mai

Può riferire gli effetti indesiderati che si verificano con un prodotto di Mylan Pharma GmbH / MEDA Pharma GmbH / MEDA Pharmaceuticals Switzerland GmbH al nostro team Farmacovigilanza all'indirizzo pv.switzerland@mylan.com, Fax: 041 768 48 11. Questo non sostituisce tuttavia l'obbligo di comunicazione alle autorità come da LATer.

KAL.0716.055.5.0119.300
