# Supplementary material for: Risk Factors, Manifestation, and Awareness of Osteoporosis among Patients of Various Specialists in Switzerland: Results of a National Survey
Source: Healthcare (Basel). 2022 Feb 3;10(2):295. doi: 10.3390/healthcare10020295 (PMC8871550; doi:10.3390/healthcare10020295)
Supplement: Supplementary file 1 [file healthcare-10-00295-s001.zip › S6.pdf]

QUESTIONNAIRE SUR LA SANTÉ OSSEUSE EN SUISSE

Chère doctoresse, cher docteur, merci beaucoup pour votre participation à notre enquête sur la sensibilisation à la santé osseuse en Suisse ! Veuillez répondre aux questions suivantes :

1) Spécialité :

- ☐ généraliste
- ☐ rhumatologue
- ☐ gynécologue
- ☐ orthopédiste
- ☐ endocrinologiste
- ☐ autres

2) Combien de patients atteints d'ostéoporose traitez-vous (sur l'ensemble de vos patients)?

☐ Aucun ☐ < 20 % ☐ 20–50 % ☐ > 50 %

3) Quelle procédure suivez-vous, si vous recevez un patient présentant une fracture prévalente ?

(Plusieurs réponses possibles)

- ☐ Aucune mesure
- ☐ Utilisation d'outils d'évaluation des fractures (FRAX, TOP-TOOL, par exemple)
- ☐ Mesure de la densité osseuse
- ☐ Radiographie
- ☐ Renvoi vers un spécialiste
- ☐ Prescription d'un complément de calcium
- ☐ Prescription d'un complément de vitamine D
- ☐ Prescription conjointe de compléments de calcium / vitamine D
- ☐ Questions sur les habitudes alimentaires et le mode de vie

4) Pourquoi traitez-vous/ne traitez-vous pas les patients atteints d'ostéoporose ?

(Plusieurs réponses possibles)

- ☐ Je me sens capable de les traiter en toute confiance
- ☐ Je souhaite préserver la santé du patient
- ☐ Je ne suis pas formé(e) pour traiter l'ostéoporose
- ☐ Je les renvoie vers des spécialistes
- ☐ Je pense qu'il est trop coûteux de les traiter moi-même

5) Dans quelle mesure les maladies suivantes occupent-elles une place importante dans votre pratique quotidienne ?

**Diabète**	importante	0	1	2	3	4	5	pas importante
**Ostéoporose**	importante	0	1	2	3	4	5	pas importante

6) Sur la base de quels critères effectuez-vous un examen concernant l'ostéoporose ?

(Plusieurs réponses possibles)

- ☐ Critères cliniques
- ☐ Dépistage (p. ex. FRAX, TOP-TOOL)
- ☐ À l'initiative du patient
- ☐ Carence nutritionnelle

7) Dans quels cas prescrivez-vous un complément de calcium / vitamine D ?

(Plusieurs réponses possibles)

- ☐ Carence nutritionnelle
- ☐ Traitement complémentaire
- ☐ Jamais
- ☐ Je suis mon intuition
- ☐ Carence en calcium / vitamine D

Vous pouvez signaler les réactions indésirables à un produit Mylan Pharma GmbH / MEDA Pharma GmbH / MEDA Pharmaceuticals Switzerland GmbH auprès de notre équipe de Pharmacovigilance par e-mail à pv.switzerland@mylan.com Fax: 041 768 48 11. Cela ne remplace cependant pas l'annonce obligatoire auprès des autorités selon LPTh.

KAL.0716.055.5.0119.300
